# The development of chronic diuretic resistance can be predicted during a heart-failure hospitalization. Results from the REDIHF registry

**DOI:** 10.1371/journal.pone.0240098

**Published:** 2020-10-02

**Authors:** Zorba Blázquez-Bermejo, Nuria Farré, Marc Llagostera, Pedro Caravaca Perez, Laura Morán-Fernández, Aleix Fort, Javier De-Juan, Sonia Ruiz, Juan F. Delgado

**Affiliations:** 1 Cardiology Department, Hospital Universitario 12 de Octubre, Madrid, Spain; 2 Cardiology Department, Hospital del Mar, Barcelona, Spain; 3 Biomedical Research Group on Heart Disease (GREC), Hospital del Mar Medical Research Group (IMIM), Barcelona, Spain; 4 Department of Medicine, Universidad Autónoma de Barcelona, Barcelona, Spain; 5 CIBER de Enfermedades Cardiovasculares (CIBERCV), Madrid, Spain; 6 Faculty of Medicine, Universidad Complutense de Madrid, Madrid, Spain; Scuola Superiore Sant’Anna, ITALY

## Abstract

**Introduction:**

Diuretic resistance (DR) is a common condition during a heart failure (HF) hospitalization, and is related to worse prognosis. Although the risk factors for DR during a HF hospitalization are widely described, we do not know whether the risk of chronic DR could be predicted during admission.

**Material and methods:**

We conducted a multicenter, prospective observational study between July 2017 and July 2019. All patients admitted for acute HF with intravenous diuretic treatment and at least one criterion of congestion on admission were invited to participate. Patients on renal replacement therapy, under intravenous diuretic treatment for >72 hours before screening and those who were unable to sign the informed consent were excluded. We monitored decongestion (physical exam, hemoconcentration, NTproBNP change and lung ultrasound) and DR (diuresis and weight loss per unit of 40mg furosemide and fractional excretion of sodium) on the fifth day of admission. Chronic DR was evaluate two months after hospitalization and was defined as persistent signs of congestion despite ≥80 mg furosemide per day. We compared variables from the hospitalization between patients with and without chronic DR. A multivariate logistic regression analysis was conducted to find predictors of chronic DR.

**Results:**

A total of 105 patients were included in the study. Mean age was 74.5±12.0 years, 64.8% were male and mean LVEF was 46±17%. In the two months follow-up, five patients have died and one patient has had a heart transplant. Of the 99 remaining patients, 21 patients (21.2%) had chronic DR. The dose of furosemide before admission and the decrease in NT-proBNP ≤30% during admission were predictors of chronic DR in the multivariate analysis.

**Conclusions:**

We can predict during a HF hospitalization which patients will develop chronic DR. The dose of furosemide before admission and the change in NT-proBNP are independent predictors of chronic DR.

## Introduction

Most of the symptoms related to acute heart failure (HF) are caused by congestion, with diuretics as the main treatment. However, diuretic resistance (DR) is a common condition during a HF hospitalization, and is related to increase readmission and mortality [1]. Generally speaking, there is DR when symptoms and signs of congestion persist even with increasing diuretic dose, but in chronic HF the definition of persistent congestion even with furosemide daily dose of ≥ 80mg has been commonly used [2, 3]. Although the risk factors for DR during a HF hospitalization are widely described, we do not know whether the risk of chronic DR could be predicted during admission. The aim of this study was to identify the prevalence and the main predictors of chronic DR after a HF hospitalization.

## Material and methods

The REsistance to DIuretic in Heart Failure (REDIHF) registry is an observational, prospective, and multicenter study, which analyses different aspects of DR in patients hospitalized for HF. In the present study, all patients admitted for acute or chronic decompensated HF in the Cardiology Department of the two participant hospitals between July 2017 and July 2019 were screened for eligibility. The inclusion criteria were age >18 years, a NT-proBNP >600 pg/mL or >1000 pg/mL in atrial fibrillation, the need for intravenous diuretic treatment and at least one criterion of congestion on admission (jugular ingurgitation, lung crackles, ascites, edema of the lower limbs, or pleural effusion on chest X-ray or lung ultrasound). Patients on renal replacement therapy, under intravenous diuretic treatment for >72 hours before screening and those who were unable to understand and sign the informed consent were excluded. One physician of the investigation team reviewed the patients admitted in the Cardiology Department everyday (except on weekends) in each hospital, and offered to participate to those who met de inclusion criteria. A total of 105 patients met the inclusion criteria, agreed to participate, and therefore enrolled in the study. Every patient gave written informed consent. The study was approved by the hospital’s ethics committees and complied with the Declaration of Helsinki and the Declaration of Istanbul.

At admission we collected demographic data, past medical history, previous medical treatment, physical examination, blood test with NT-proBNP, and electrocardiography and echocardiography data. Relevant demographic details are shown in Table 1. We consider the sample is representative of the population admitted for HF in most of the European countries because our inclusion and exclusion criteria were not excessively strict; besides, the demographic characteristics of our patients were quite similar to the patients included in the HF registry of the European Society of Cardiology [4]. The diuretic dose and the rest of the medical treatment were left to the discretion of treating physicians. On the fifth day of admission, we assessed decongestion by physical examination, hemoconcentration, change in NT-proBNP, and lung ultrasound (number of B-lines in 28 spaces and pleural effusion). Likewise, on the fifth day we evaluated DR in the acute phase with the fractional excretion of sodium, and with the weight loss or diuresis from admission per unit of 40mg furosemide (diuretic efficiency). An on-site visit was carried out two months after inclusion. We defined chronic DR as the persistence in this visit of at lest one criterion of congestion (jugular ingurgitation, lung crackles, ascites or edema of the lower limbs) despite ≥ 80mg of furosemide per day.

**Table 1:** Demographic data.

Age (years)	74.5 ± 12.0
Male sex	64.8%
Diabetes mellitus	42.9%
Arterial hypertension	85.7%
Dyslipidemia	66.7%
Body mass index (kg/m2)	28.9 ± 5.3
De novo heart failure	37.1%
Significant coronary artery disease	34.3%
Moderate-to-severe valvular heart disease	41.0%
Atrial fibrillation	61.9%
LVEF (%)	46 ± 17
NT-proBNP at admission (pg/mL)	4875 (2537–11093)
Glomerular filtration rate at admission (mL/min/1.73m2)	55 ± 21
Chronic obstructive pulmonary disease	23.8%
Cerebrovascular disease	8.6%
Peripheral vascular disease	6.7%

Categorical variables are expressed as percentage and quantitative variables as mean ± standard deviation, except for NT-proBNP that is expressed as median (interquartile range). LVEF: Left ventricular ejection fraction.

### Statistical analysis

Discrete variables were expressed as a proportion and continuous variables as a mean (standard deviation) or as a median (interquartile range). Patients who did not complete the two months follow-up were excluded from the analysis. In order to identify the risk factors that would predict DR after a HF admission, we compared variables from the hospitalization between patients with and without chronic DR. Differences between groups were analyzed using the Student’s t-test for continuous variables, and χ^2^ test or Fisher’s exact test for discrete variables. A multivariate logistic regression analysis, including all the variables with p<0.1 in the univariate analysis, was performed using a step-wise forward manner to identify independent predictors of chronic DR. Odds ratios (OR) and 95% confidence intervals (CI) were calculated for each risk factor. We performed a logistic model including all independent risk factors. Receiver-operating characteristic (ROC) curve analysis was used to evaluate the area under the ROC curve (AUC) of the logistic model to predict chronic DR. A p value <0.05 was considered significant. The statistical analysis was performed using Stata software (V.14.0, Stata Corporation).

## Results and discussion

**Fig 1:**
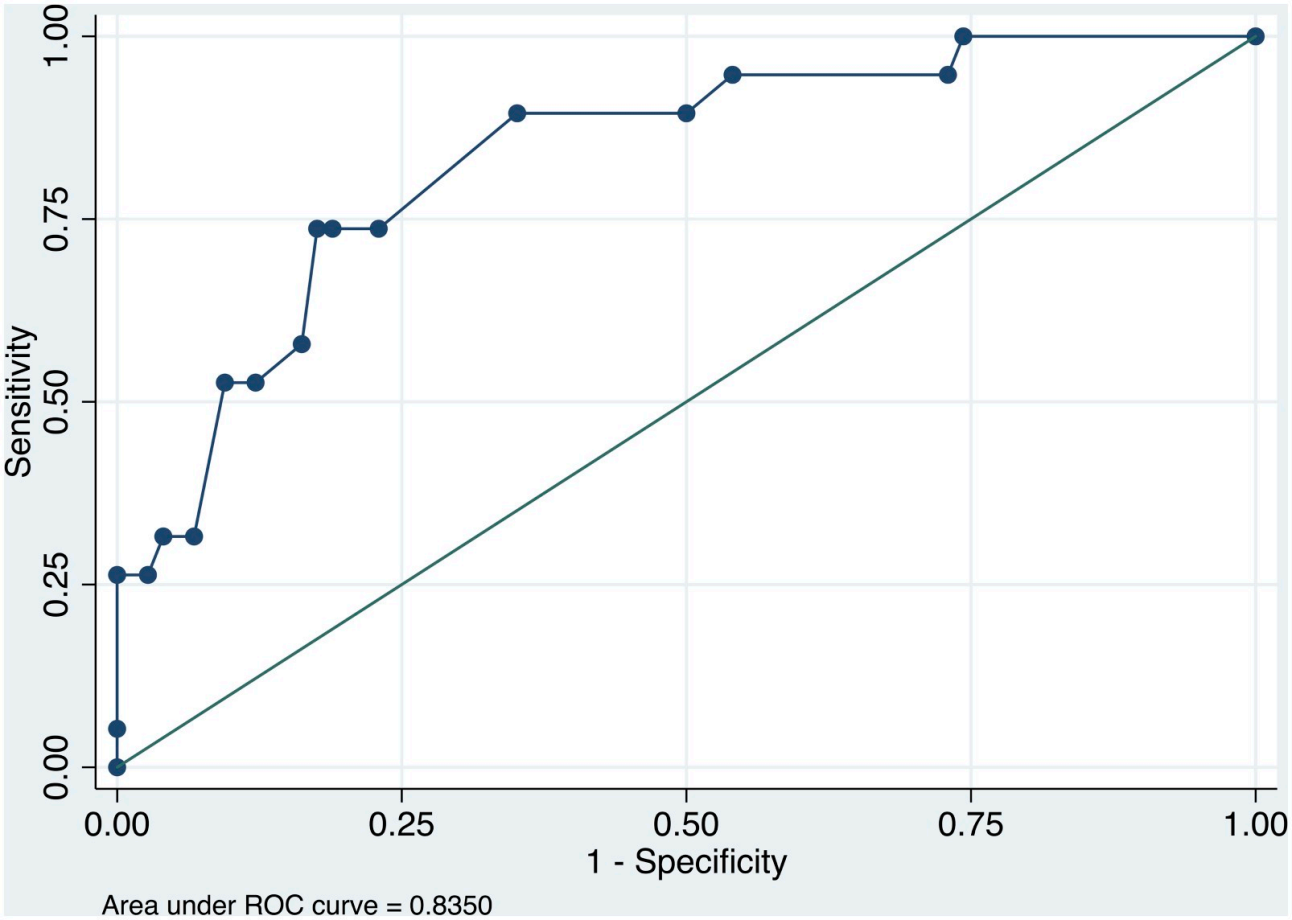
ROC curve of the model to predict chronic diuretic resistance.

A total of 105 patients were included during de study period. In the two months follow-up, five patients have died (two of them during admission) and one patient has had a heart transplant. Of the 99 remaining patients, 21 patients (21.2%) had chronic DR. In this group, in the two months follow-up, the mean furosemide dose was 129±56mg vs 69±55mg in the no-DR patients (p<0.001), and 23.8% were receiving thiazide diuretics vs 10.3% (p = 0.103). Furthermore, with regard to the hospitalization, patients with chronic DR had higher prevalence of moderate-to-severe valvular heart disease, chronic HF, greater dose of furosemide before admission, as well as lower increase in hematocrit, lower proportion of >30% decrease in NT-proBNP and lower diuretic efficiency. The dose of furosemide before admission and the decrease in NT-proBNP ≤30% during admission were predictors of chronic DR in the multivariate analysis (Table 2). In addition, the logistic model that included these two variables had an AUC of 0.835 to predict chronic DR (Fig 1).

**Table 2:** Differences during hospitalization between patients with and without chronic diuretic resistance.

Hospitalization variables	Diuretic resistance in chronic phase (n = 21)	No diuretic resistance in chronic phase (n = 78)	p value	Multivariate analysis OR (CI 95%)
Age (years)	70.1±14	76.0±11	0.041	NS
Male sex	15 (71.4%)	47 (60.3%)	0.348	-
Diabetes mellitus	7 (33.3%)	35 (44.9%)	0.342	-
Arterial hypertension	18 (85.7%)	67 (85.9%)	0.983	-
Dyslipidemia	15 (71.4%)	53 (67.9%)	0.760	-
De novo heart failure	3 (14.3%)	36 (46.2%)	0.011	NS
Significant coronary artery disease	7 (33.3%)	28 (35.9%)	0.827	-
Moderate-to-severe valvular heart disease	14 (66.7%)	27 (34.6%)	0.008	NS
Atrial fibrillation	14 (66.7%)	46 (59.0%)	0.522	-
LVEF (%)	46±19	45±16	0.760	-
Dose of furosemide before admission (mg)	98±56	41±45	<0.001	1.022 (1.010–1.034)
Thiazide before admission	3 (14.3%)	15 (19.2%)	0.756	-
Beta-blocker before admission	18 (85.7%)	55 (70.5%)	0.160	-
ACE inhibitor or ARB before admission	9 (42.9%)	46 (59.0%)	0.187	-
Sacubitril/Valsartan before admission	2 (9.5%)	5 (6.4%)	0.638	-
MRA before admission	8 (38.1%)	18 (23.1%)	0.165	-
SGLT2 inhibitor before admission	0 (0%)	2 (2.6%)	1.000	-
Systolic blood pressure at admission (mmHg)	118±22	127±20	0.080	NS
Diastolic blood pressure at admission (mmHg)	66±15	69±15	0.297	-
Heart rate at admission (bpm)	84±22	81±20	0.593	-
Glomerular filtration rate at admission (mL/min/1.73m^2^)	51±18	57±22	0.271	-
Creatinine at admission (mg/dL)	1.49±0.63	1.30±0.58	0.189	-
Urea at admission (mg/dL)	73±41	61±31	0.176	-
Sodium at admission (mmol/L)	140±4	141±4	0.368	-
ALT at admission (U/L)	41±61	34±60	0.638	-
AST at admission (U/L)	34±18	43±107	0.703	-
GGT at admission (U/L)	85±61	66±60	0.201	-
Bilirubin at admission (mg/dL)	1.14±0.94	0.92±0.54	0.314	-
Albumin at admission (g/dL)	4.0±0.3	3.9±0.4	0.618	-
TSH at admission (μUI/mL)	3.5±1.7	2.1±1.7	0.002	NS
Decrease in NT-proBNP >30%	7 (36.8%)	54 (71.1%)	0.005	0.223 (0.066–0.748)
Change in hematocrit on the 5^th^ day (%)	-0.98±3.97	1.23±3.51	0.017	NS
FENa on the 5^th^ day (%)	1.47±1.3	1.41±1.8	0.888	-
B-lines in lung ultrasound on the 5^th^ day	4.8±5.5	3.3±5.9	0.345	-
VAS of dyspnea on the 5^th^ day (0–10)	3.1±3.2	1.7±1.9	0.081	NS
Change in weight per unit of furosemide (kg)	−0.27±0.33	−0.45±0.59	0.188	-
Diuresis per unit of furosemide (L)	0.74±0.40	1.05±0.62	0.010	NS

Categorical variables are expressed as number (percentage) and quantitative variables as mean ± standard deviation. OR: Odds ratio; CI: Confidence interval; NS: Non-significant difference; LVEF: Left ventricular ejection fraction; ACE: Angiotensin-converting enzyme; ARB: Angiotensin II receptor blocker; MRA: Mineralocorticoid receptor antagonist; SGLT2: Sodium-glucose contransporter-2; ALT: Alanine transaminase; AST: Aspartate transaminase; GGT: Gamma-glutamyl transferase; TSH: Thyroid-stimulating hormone; FENa: Fractional excretion of sodium; VAS: Visual analogue scale.

The pathophysiology of DR in HF is complex and not fully known, but it relates to a combination of factors including renal disease, the activation of the renin-angiotensin-aldosterone system and the escape mechanisms in the kidney such as the “braking phenomenon” [5, 6]. Other mechanisms involved may be the decreased intestinal absorption of oral diuretics, due to the edema in the intestinal wall, or the presence of hypoalbuminemia, as the majority of diuretics are bound to albumin in serum [7, 8]. Many studies have described risk factors of DR such as diabetes mellitus, ischemic heart disease, renal disease or arterial hypotension; however, these studies have assessed DR in acute HF [1, 9–13]. Moreover, most of the data has been obtained from post-hoc analyses of clinical trials, which included very specific patients, and therefore its extrapolation to the real-world population with HF may be problematic. Elderly patients, or those with preserved LVEF or comorbidities were frequently excluded in these trials.

This study shows a high prevalence of chronic DR after HF admission in a real-world population. As was noted in other studies, patients with chronic DR have greater HF readmissions and mortality [3]. On the bases of our results we can predict chronic DR during a HF hospitalization, which is highly useful for deciding the better management after discharge. On the one hand, in those patients at high risk for chronic DR we could avoid insufficient dosing of loop diuretic, add a thiazide diuretic or educate the patient about flexible diuretic regimen. On the other hand, the identification of these patients would allow us to follow them up very closely, for example planning an early visit after discharge, organize a structured follow-up by a HF specialist nurse or include them in telemedicine programs [14–16].

The independent predictors of chronic DR, oral dose of furosemide before admission and change in NT-proBNP during admission, have shown prognostic value in previous studies [17, 18]. On one side, it is no surprising that higher previous dose of furosemide predicts worse prognosis and, in our study, chronic DR. It may identify a more severe HF, as can be concluded from its association to a higher prevalence of chronic HF, as well as less hemoconcentration and less diuretic efficiency during hospitalization. On the other hand, NT-proBNP is a well-studied prognostic factor in HF and it usually decreases in response to HF treatment. The inability to significantly decrease NT-proBNP during admission may reflect an insufficient HF treatment or, more likely, a more severe HF.

However, several limitations of this study are worth noting. First of all, despite being a representative sample of the acute HF population, the number of patients included was relatively small. This may explain why renal insufficiency, diabetes mellitus or arterial hypotension, known risk factors of DR, was not associated with chronic DR in our patients. Second, the model to predict chronic DR should be validated in a different patient cohort, thus we consider our work a hypothesis-generating study. Third, urinary sodium and FENa are used to evaluate diuretic resistance in HF. These parameters have been classically used to assess tubular function and are essential to differentiate between functional and parenchymal nephropathy. Unfortunately, for logistical reasons our protocol did not include FENa at admission. Finally, although DR is a common problem in HF, its pathophysiology is complex, multifactorial and dynamic; this makes it difficult to exclude confounding factors in chronic DR such as medical interactions, diuretic effect from other drugs or significant variations in DR over time.

## Conclusions

In conclusion, our results suggest that the development of chronic DR can be predicted during a HF hospitalization. The dose of furosemide before admission and the change in NT-proBNP are independent predictors of chronic DR. Identifying these patients during admission could serve to individualize diuretic treatment as well as follow-up after discharge.
